# Study of antagonistic effects of *Lactobacillus* strains as probiotics on multi drug resistant (MDR) bacteria isolated from urinary tract infections (UTIs)

**Published:** 2014-03

**Authors:** Atiyeh Naderi, Roha Kasra-Kermanshahi, Sara Gharavi, Abbas Ali Imani Fooladi, Meghdad Abdollahpour Alitappeh, Parvaneh Saffarian

**Affiliations:** 1 Division of Microbiology, Department of Biology, Faculty of Sciences, Alzahra University, Tehran, Iran; 2 Applied Microbiology Research Center, Baqiyatallah University of Medical Sciences, Tehran, Iran; 3 Department of Pharmaceutical Biotechnology, Pasture Institute, Tehran, Iran; 4 Department of Bacteriology, Faculty of Medical Sciences, Tarbiat Modares University, Tehran, Iran

**Keywords:** Antibiotic resistance, * Lactobacillus*, Pathogenic bacteria, Probiotic, Urinary tract infection

## Abstract

***Objective(s):*** Urinary tract infection (UTI) caused by bacteria is one of the most frequent infections in human population. Inappropriate use of antibiotics, often leads to appearance of drug resistance in bacteria. However, use of probiotic bacteria has been suggested as a partial replacement. This study was aimed to assess the antagonistic effects of *Lactobacillus *standard strains against bacteria isolated from UTI infections.

***Materials and Methods:*** Among 600 samples; those with ≥10,000 cfu/ml were selected as UTI positive samples. *Enterococcus *sp., *Klebsiella pneumoniae*, *Enterobacter *sp., and *Escherichia coli* were found the most prevalent UTI causative agents. All isolates were screened for multi drug resistance and subjected to the antimicrobial effects of three *Lactobacillus* strains by using microplate technique and the MICs amounts were determined. In order to verify the origin of antibiotic resistance of isolates, plasmid curing using ethidium bromide and acridine orange was carried out.

***Results:*** No antagonistic activity in *Lactobacilli* suspension was detected against test on *Enterococcus *and* Enterobacter *strains and* K. pneumoniae,* which were resistant to most antibiotics. However, an inhibitory effect was observed for *E. coli* which were resistant to 8-9 antibiotics. In addition, *L. casei* was determined to be the most effective probiotic. Results from replica plating suggested one of the plasmids could be related to the gene responsible for ampicillin resistance.

***Conclusion:*** Treatment of *E. coli* with probiotic suspension was not effective on inhibition of the plasmid carrying hypothetical ampicillin resistant gene. Moreover, the plasmid profiles obtained from probiotic-treated isolates were identical to untreated isolates.

## Introduction

Urinary tract infection (UTI) is the second most common infection among patients being presented to medical practices (1). Approximately 150 million people in world are being infected with UTI per year, either complicated or uncomplicated form (2). UTI may only happen in the lower urinary tract or in both upper and lower tracts. Clinical syndromes include: dysuria, frequency, occasional suprapubic tenderness in cystitis and urethritis, flank pain, fever, and urgency in pyelonephritis (3).

The most common organisms that cause UTI are limited numbers of *E. coli *serotypes which have increased adhesion, colonization and tissue invasion properties compared to nonpathogenic bacteria. These features are mediated by pili, agents that increase resistance to bactericidal activities and mediators of invasiveness (4). Another common pathogen in UTI is *Klebsiella pneumoniae *(*K. pneumoniae*) (5) that stands in second level after Escherichia coli (*E. coli*). However*, Enterobacter *spp. (6) and some Gram positive bacteria like *Enterococcus *spp.*, *are also involved in UTI (7). 

Antibiotics can be effective against bacteria through five major mechanisms of action (8): interference with cell wall synthesis (9, 10); inhibition of protein synthesis (9, 11); prevention of nucleic acid synthesis (9); suppression of a metabolic pathway (9); and disorganizing the cell membrane (12, 13). The main problem with the use of antibiotics is that bacteria are able to evolve thus they can acquire resistance against antibiotics via several biochemical aspects such as antibiotic inactivating, target modification, changes in efflux pump and outer membrane (OM) permeability and by-pass the target along with some genetic aspects such as mutation and horizontal gene transfer (14-16). Probiotic bacteria, such as *Lactobacillus strains are* most commonly group of microorganisms utilized for treatment of many infectious diseases in humans (17, 18). These bacteria are well-known since they contain many beneficial properties to control pathogenic microorganism’s ability. These properties include adherence to cells, reduction of pathogenic bacterial adherents and co-aggregation, and production of organic acids which antagonize pathogenic microorganisms. Moreover, they are nonpathogenic (19, 20). In addition, there have been many reports on production of bacteriocin by *Lactobacilli* bacteria (21, 22). It is known that the antagonistic activity of such bacteria is to inhibit a large number of enteric and urinary tract bacterial pathogens (23-25). Lactic acid bacteria (LAB) are a group of Gram positive, non- respiratory, non-spore forming coccoid or rod shaped probiotics which produce lactic acid as major end product by fermentation of carbohydrates, and display numerous antimicrobial activities. This is mainly due to the production of organic acids and other compounds such as bacteriocins and antifungal peptides (26, 27). The aim of this study was to assess antagonistic activity of three *Lactobacillus* standard strains as probiotic, against multi drug resistant bacteria isolated from urinary tract infections.

## Materials and Methods

***Bacteria strains and media***

Probiotics *Lactobacilli* (*L. acidophilus *ATCC3456, *L. casei *ATCC 39392 and *L. rhamnosus *ATCC 7469) and standard strains *Staphylococcus aureus *(*S. aureus*) ATCC 25923 and *E. coli* ATCC25922, as multi drug resistant bacteria; as well as De Man-Rogosa-Sharpe (MRS) broth and agar (Merck), Luria-Bertani (LB), CHROM agar (FRANCE), Tryptic Soya broth (TSB), Mueller Hinton agar (Merck), Methylen blue milk, KF agar (Merck), MacConkey Agar (Merck), and Triple Sugar Iron agar (TSI) (Merck) were used in this research.

***Isolation and Identification of bacteria***

Almost 600 urine samples were collected over 6 months and submitted to the microbiological laboratory of Applied Microbiology Research Center, Iran. The majority of samples were midstream urine specimens and the rest included catheterized urine samples and suprapubic aspirates. Culture was done by the calibrated loop technique delivering 10 µl of urine and plated on CHROM agar plates (as differential media) for primary isolation and rapid identification of urinary pathogens, and incubated at 37°C for 24 hr. This identification culture method was confirmed by culture of urine samples on MacConkey agar and blood agar. Biochemical tests were also carried out (3, 28, 29). 

***Susceptibility testes***

Antibiotic susceptibility test was performed by disk diffusion method in Mueller-Hinton agar (30). The results were according to guidelines of the National Committee for Clinical Laboratory Standards (31). The tested antibiotics were as follows: cephalexin (CN), ceftriaxone (CRO), ampicillin (AM), cefotaxime (CTX), trimethoprim-sulfamethoxazole (SXT), tetracycline (TE), nalidixic acid (NA), ciprofloxacin (CP), amikacin (AN), gentamicin (GM), nitrofurantoin (FM), doxycycline (D), erythromycin (E), penicillin (P) and vancomycin (V). The resistant patterns of *E. coli* strains were similar to those resistant to 8 to 9 antibiotics. Ten isolates were selected randomly for next steps. The minimal inhibitory concentrations (MICs) for 10 *E. coli* isolates with highest resistance to three antibiotics: AM, Cp and SXT- which are the drugs of choice in the treatment of UTI- were determined by broth microdilution method as described in the NCCLS guidelines (Table 1) before and after treatment with probiotics. Quality control (QC) was performed concurrently during the processing of specimens using standard strains including *S. aureus *ATCC 25923 and *E. coli *ATCC 25922.

***Assessment of Lactobacillus bacteria antagonistic activities as a probiotic***

Cell free supernatants (CFs) from *Lactobacilli* were obtained and their inhibitory activity against isolated uropathogenic bacteria were assayed by agar well diffusion method (20). *Lactobacilli *were grown in De Man-Rogosa-Sharpe (MRS) broth at 37°C in 5% CO_2_ for 48 hr; then cultures were centrifuged at 1500 rpm for 15 min and the supernatants were decanted. Supernatants were sterilized using 0.22 μm Millipore filters. Prior to sterilization, half of the supernatants were neutralized to pH 7.0±0.01 by NaOH (1 mol/lit). Furthermore, each test pathogen was cultured overnight in Tryptic Soy Broth (TSB). Then growth bacteria were streaked on the surface of Mueller-Hinton agar with a turbidity equivalent to 0.5 McFarland standards. A sterile Pasture pipette was used to make well (6 mm diameter) on the surface of streaked agar. Small drop of MRS agar was put inside the edge of each well before adding CFs.

**Figure 1:**
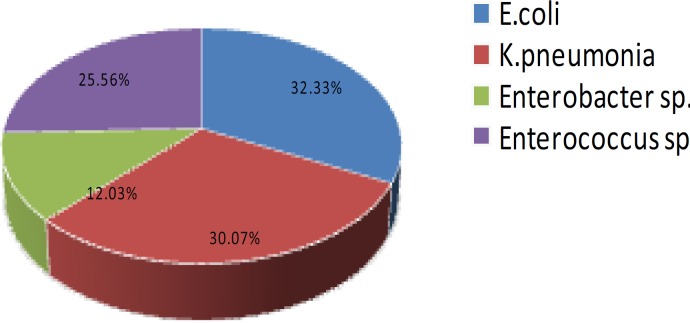
Frequency of all bacterial strains isolated from urine samples

Both pH-adjusted and -unadjusted supernatants from the cultures of *Lactobacilli *were placed in the well (100 µl). The plates were incubated for 5 hr at room temperature and then for 24 hr at 37°C. A clear inhibition zone of 6 mm in diameter was defined as positive result. Moreover, the MIC of the supernatant was determined according to Wikler *et al*, 2006 method (30) with some differences: the highest concentration was 250 l/ml and the lowest 0.5 l/ml, in addition that the supernatant was directly added to the wells.

***Examination of the genetic origin of resistance in E. coli strains by plasmid curing method***

Since ethidium bromide was used as a removal agent in this study, obtaining a defined concentration of ethidium bromide whereby it would be effective as well as the bacteria can grow is necessary. For this purpose, the amount of MIC was determined for ethidium bromide according to Wikler *et al* (30). Subsequently, 200 l of overnight-culture of bacterium was added to the LB broth medium. In the next step, ethidium bromide was added to the media in sub-MIC concentration and the tubes were incubated for 18-24 hr at 37C. Then the tubes were transferred to the LB broth medium and were incubated at 37C for 3-4 hr until the turbidity of 0.5 McFarland standard was achieved. Afterward, 0.1 ml of the suspension containing 1.5 10^8^ bacterium per ml was transferred to the nutrient agar medium and was incubated for 24 hr at 37C. After incubation, about 100 colonies appeared on the plate. Microtiter plates with 96 wells were used for isolation of bacteria which had lost their resistance. Two hundred microliters of the nutrient broth medium was added to each well and the colonies were transferred to the wells (one colony per well). Microtiter plates were incubated at 37C for 4-5 hr. Subsequently, simultaneous inoculation of microbial suspensions was performed using a multiple-inoculating machine from wells to the plates containing medium and antibiotics and also to the nutrient agar plates as blank. The transferred plates were incubated at 37C for 24-48 hr. Finally, colonies with no growth on the screening plates but grew on the blank plates were considered as mutant colonies (32). 

**Figure 2:**
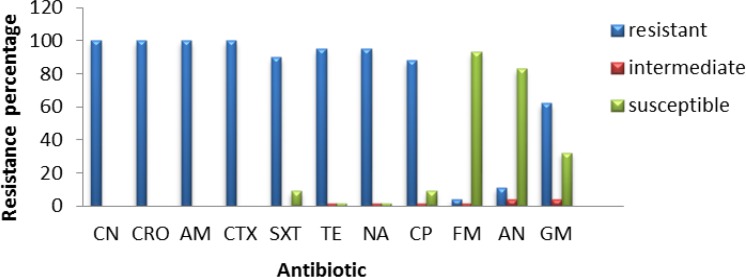
Frequency of antibiotic resistance in isolated* Escherichia coli* from urine samples

**Figure 3:**
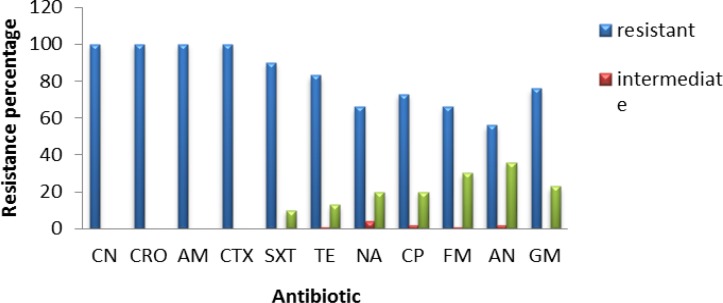
Frequency of antibiotic resistance in isolated *Klebsiella pneumoniae* from urine samples

**Figure 4:**
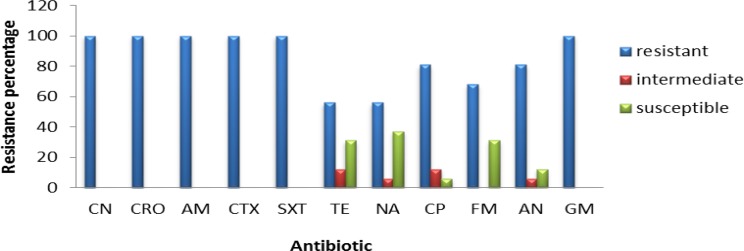
Frequency of antibiotic resistance in *Enterobacter* strains isolated from urine samples

**Figure 5:**
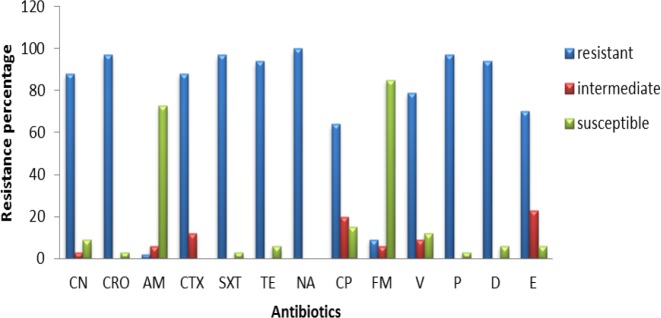
Frequency of antibiotic resistance in *Enterococcus* strains isolated from urine samples

***Study on the effect of lactobacilli culture supernatant on plasmid deletion in E. coli isolates***

For this purpose, the MIC of lactobacilli cells were determined as explained above. Then bacteria were cultured in sub-MIC (1/2 MIC) concentrations of the supernatant. After incubation at 37C for 12-18 hr, bacteria were cultured in LB broth medium and were incubated until achieving 0.5 McFarland standards. The next steps were carried out as described earlier for the ethidium bromide.

***Molecular evaluation***

In order for confirming replica plating method, plasmid extraction was performed by alkaline lysis method (33). Plasmids were separated by electrophoresis on 0.8% (wt/vol) agarose gel for 3 hr at 60 V using TAE (Tris-acetate-EDTA) buffer (pH, 8.0) and visualized by ethidium bromide staining (Sigma, St. Louis, MO) solution (0.5 mg/ml) under short wave UV light. The resultant gel containing plasmid pattern, was scanned and results were prepared with that of replica plating method.

## Results

Among 600 analyzed samples, those with 10^4 ^CFU/ml were chosen as positive UTI. Eventually, four bacterial species with high frequency were detected. The overall species distribution has been shown in Figure 1. More than 87% of the isolated bacteria belonged to the entrobacteriaceae. *E. coli* was the most frequently isolated species between our UTI samples (32.33%) followed by *K. pneumoniae* (30.07%) (Figure 1).

***Antimicrobial susceptibility testing***

Nearly 133 bacterial samples were analyzed to determine antibiotic susceptibility. Results of the antibiotic susceptibility testing are shown in Figures 2, 3, 4 and 5 for *E. coli*, *K. pneumoniae*, *Enterobacter *sp*.*, and *Enterococcus,* respectively. All *E. coli* isolates (100%) obtained from urine samples, showed resistance to CN, CRO, AM and CTX antibiotics. The most susceptibility of isolated strains was against FM and AN (Figure 2).

All *K. pneumoniae* isolates showed resistance to CN, CRO, AM, and CTX antibiotics and almost 90% of them were resistant to SXT and TE, with 50-70% of strains being resistant to other antibiotics (Figure 2). Moreover, 16 isolated strains belonged to *Enterobacter *sp*.,* all of them being resistant to Cefalexin, CRO, AMP, CTX, SXT and GM antibiotics, and almost 50-80% of them were resistant to other antibiotics (Figure 3).

**Figure 6:**
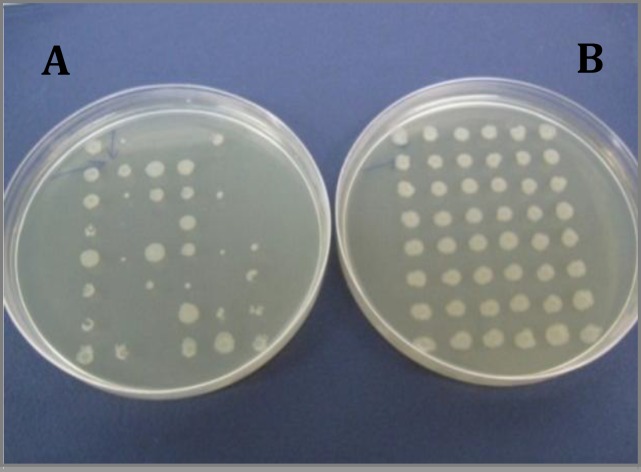
Isolation of ampicillin sensitive mutant strains created by treating the strain number 5 with Ethidium bromide and acridine orange using replica plating method. Plate A: plate without ampicillin; plate B: plate containing ampicillin

**Figure 7:**
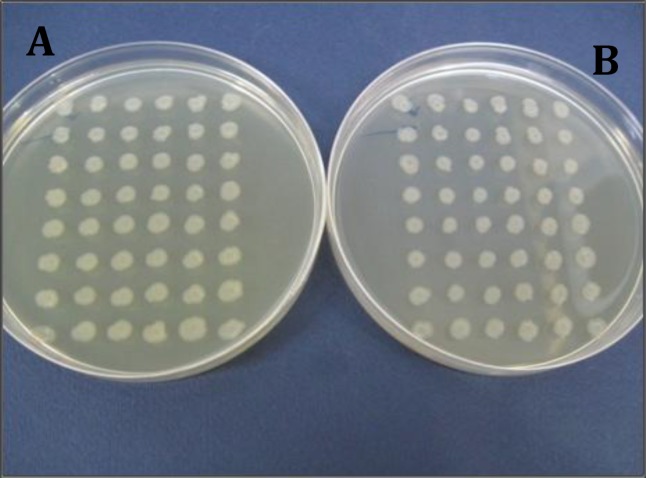
Isolation of ciprofloxacin sensitive mutant strains created by treating the strain number 5 with Ethidium bromide and acridine orange using replica plating method. Plate A: plate without ciprofloxacin; plate B: plate containing ciprofloxacin

**Table 1:** Initial and final concentration of antibiotics according to CSLI guideline

Antibiotic	Concentration range	Initial concentration	Final concentration
Ampicillin	0.5-256 µg/ml	1024 µg/ml	256 µg/ml
Ciprofloxacin	0.5-256 µg/ml	1024 µg/ml	256 µg/ml
Trimetoprim-sulfometaxazole	0.5-256 µg/ml	1024 µg/ml	256 µg/ml

All *Enterobacter *isolates were resistant to all tested antibiotics, but about 40% of them showed susceptibility to TE and NA (Figure 4). All 34 isolates of *Enterococcus,* showed resistant to NA, while almost 75-85% of them were susceptible to AM and F (Figure 5). The range of MICs for AM between *E. coli* isolates showing the highest resistance, were 256, 128 and 64. According to CLSI breakpoints, a strain is considered resistant to AM if its MIC is 32 µg/ml or above, and it is considered resistant to CP if its MIC is 4 µg/ml or above. Furthermore, CLSI breakpoint of SXT is MIC 8.52. In this study, ranges of MICs for CP among *E. coli* isolates with high resistance were 128, 64 and 32; while MICs of SXT among all *E. coli* isolates were 64. The results of MICs in this study were similar to the antibiogram results and showed that all *E. coli* isolates were resistant to first line antibiotics of UTI treatment.

***Analysis of Lactobacillus probiotic*** ***activity***

After selection of isolated strains with high range of resistance to antibiotics and standard species *Lactobacillus, including L. acidophilus *ATCC 3456, *L. casei *ATCC 39392 and *L. rhamnosus *ATCC 7469, assessment of antagonistic activity performed by well diffusion method. No antagonistic activity for *lactobacilli* strains against *K. pneumoniae*, *Enterobacter *sp. and *Enterococcus *sp. was detected (data not shown). Antibacterial effect of cell free supernatant (CFs) of probiotic lactobacilli by well diffusion method was only observed in those *E. coli *strains with resistance to 8 or 9 antibiotics (Table 2). The most inhibition zone for *E. coli *was obtained by *L. casei* (Table 2)*.* To confirm these results, MICs of lactobacilli culture supernatants against 10 selected strains of *E. coli* with resistance to eight or nine antibiotics were determined. The amount of MIC for *L. casei* ATCC 39392 supernatant was lower than the two other species and therefore, was more effective. In addition, an indirect relationship was detected between pH values in response to acid production and the antibacterial activities of the used probiotics. 

***Examination of resistance genetic origin in E. coli strains by plasmid curing method***

Since the effect of treatment of *E. coli* strains with lactobacilli supernatants was not very considerable between the strains with patterns of resistance to eight or nine antibiotics, those strains which had larger inhibition halo were selected for molecular analyses (strains number 5 and 6). After treating the mentioned strains with ethidium bromide, the resulted mutant strains were isolated. Strain number 5 neither grew on the plates containing AM nor produced much smaller colonies than normal size (Figure 6), while strain number 6 grew normally. Both strains also grew on plates containing CP (Figure 7).

**Figure 8:**
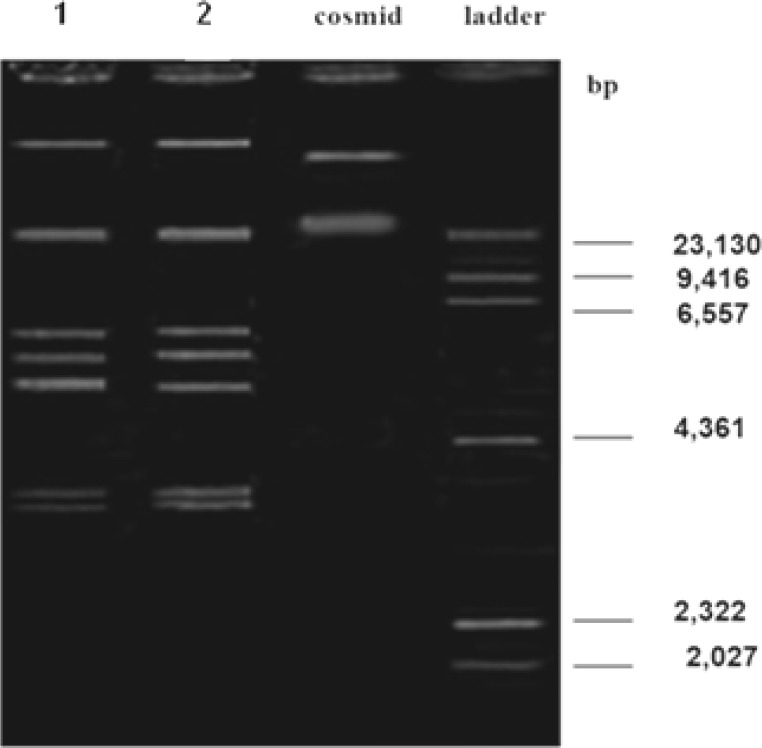
Agarose gel electrophoresis showing plasmid patterns of Lane 1: small colonies growth on plates containing amikacin after treating with ethidium bromide; Lane 2: maternal strain

**Figure 9:**
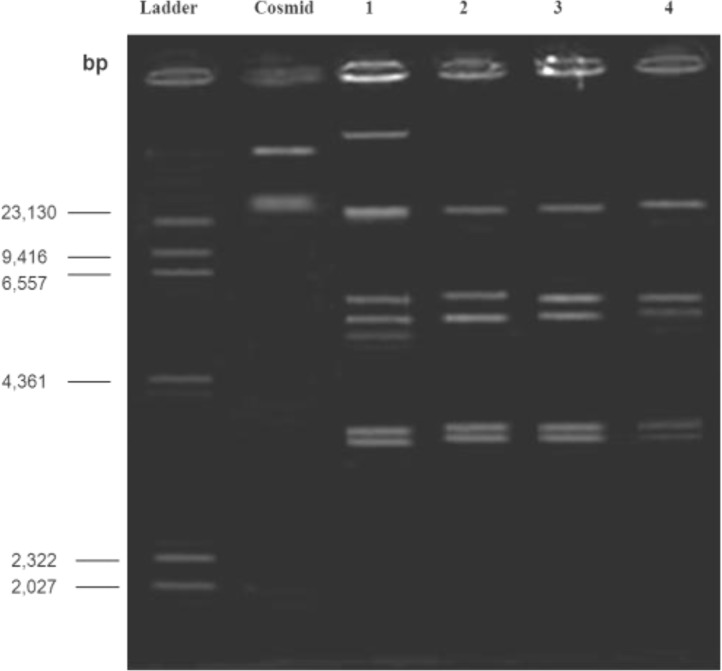
Agarose gel electrophoresis. Lane 1: plasmid patterns of the strain number 5; Lane 2: plasmid patterns of the strain number 5 after treatment with ethidium bromide; Lane 3: plasmid patterns of the strain number 5 after treatment with acridine orange; Lane 4: maternal strain

Plasmid extraction from small colonies was carried out except there was not any difference between maternal patterns (Figure 8). To confirm the results of replica plating method, plasmid pattern of strain number 5 was prepared and compared with those of wild strain; the former pattern had two bands less than the latter (Figure 9). Moreover, noteworthy that the amount of MIC for AM in mutant strain was less than the wild strain (MIC= 16 ml/ µg). Therefore, it is likely that removed bands are related to resistance to ampicillin. However, it needs more research to confirm.

***Effect of Lactobacilli culture supernatant on plasmid deletion in E. coli isolates***

**Figure 10:**
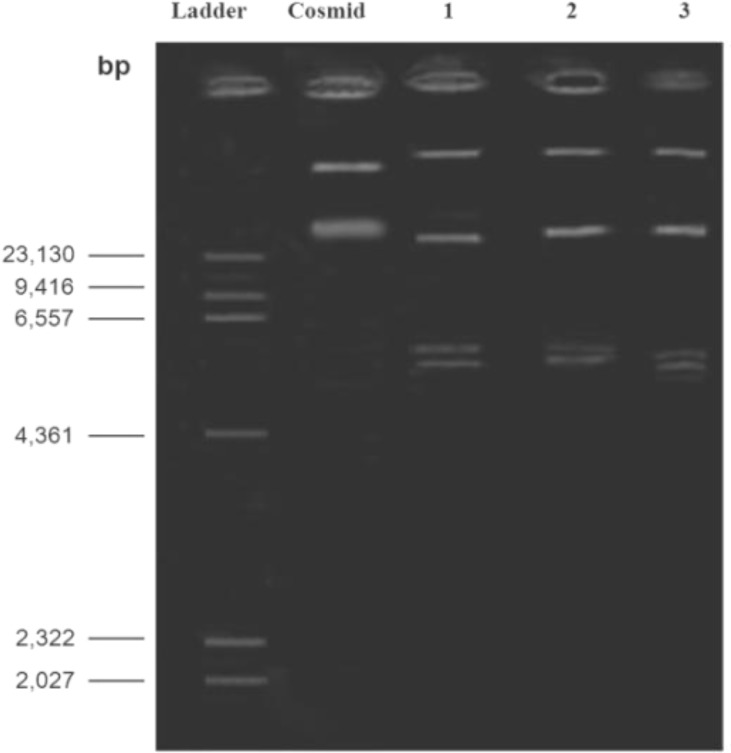
Agarose gel electrophoresis showing plasmid pattern of strain number 6. Lane 1: treated strain with *L. casei* supernatant; Lane 2: treated strain with *L. acidophilus* supernatant; Lane 3: treated strain with *L. rhamnosus* supernatant

After incubation of test bacteria in the LB media containing sub-MIC concentrations of each *lactobacillus* culture supernatants, isolation of mutant strains were carried out using replica plating method and no mutant was identified. Moreover, plasmid extraction from treated strains was performed and plasmid patterns of each sample were determined; however, no difference was observed between plasmid patterns of treated strains with that of maternal strain (Figure 10).

**Table 2:** The inhibition zone of growth for isolated *Escherichia coli *strains and standard strain *E. coli *ATCC 25923 by probiotic *lactobacilli* CFs

Cell free supernatant	Inhibition zone diameter (mm)										
*Lactobacillus *strains	*E. coli *1	*E. coli* 2	*E. coli *3	*E. coli *4	*E. coli* 5	*E. coli* 6	*E. coli* 7	*E. coli* 8	*E. coli *9	*E. coli *10	*E. coli* ATCC 25922
*L. acidophilus*	13	7.7	12	13	14	13.5	11.5	11	11.5	10	12
*L. casei*	17	11.5	12.5	13.5	14.5	13.5	11	13.5	13	12.5	12.5
*L. rhamnosus*	14.5	10	12	13	14	12.5	10	13	12	9	-

## Discussion

In recent decades, resistance to common antibiotics in the treatment of urinary tract infections caused by bacteria has been increased which has created serious problems in treatment of these diseases. Nowadays, rapid identification methods are very useful in discrimination of bacteria from urinary samples (32). In this research, chrome agar medium was used for isolation of bacterial agents in UTI. Bacterial species were initially isolated based on their color and morphology being created on chrome agar medium. Among the isolated bacteria, *E. coli*, *Klebsiella*, *Enterobacter* and *Enterococcus *were the most frequent, while the most common cause of UTI was *E. coli* with a prevalence of 33%. The lowest resistance among *E. coli* strains was against AN and FM which was determined as 11.62% and 4.64%, respectively. Therefore, these two antibiotics are suggested as appropriate antibiotics for treatment of UTI caused by *E. coli*. The *Klebsiella* strains were completely resistant to antibiotics CN, CTX, CRO and AM and the lowest resistance was related to AN, FM and NA with a resistance frequency of 56.66%, 66.66% and 66.66%, respectively; thus being suggested for treating UTIs caused by *K. pneumoniae*. Among *Enterobacter *strains*,* the lowest resistance was observed for TE and NA with the same resistance frequency of 56.25%; thus accounting as candidate for treatment of UTIs caused by *Enterobacter*. Regarding *Enterococcus*, 100% of strains were resistance to NA while the lowest resistance was observed for ampicillin; so, this antibiotic could be a candidate for treating UTIs caused by these bacteria. 

In a study it demonstrated that, very low susceptibility to first-choice agents for treatment of UTIs (Ampicillin, Amoxicillin, Clavulanic acid, CP and Cotrimoxazole) was reported, although had an acceptable susceptibility to FM and AN (34). Therefore, these antibiotics were introduced as appropriate agents for UTIs treatment which is highly similar to our results. 

Among 10 selected *E. coli* strains, only one strain showed MIC ≥ 256 for ampicillin, while MIC range for the rest of isolates were ≥ 128 and ≥ 64 being much higher than the suggested MIC for AM resistant *E. coli* strains (MIC ≥ 32) by CLSI (30). Hence, all 10 strains were reported as AM resistant. Most of the strains had the same MIC and MBC, but in three of the strains the amount of MBC was two times more than that of MIC. The amount of CP MICs for *E. coli* isolates were more than what has been suggested by CLSI (MICs≥ 4) (30). Therefore, all strains were also resistant to CP and their MBC was equal or two times more than MIC. The MIC of cotrimoxazole against all *E. coli* isolates was 64; thus, regarding the CLSI (MICs≥ 8.52), all the isolates were as well resistant to cotrimoxazole. Furthermore, their MIC was equal to MBC. 

It has been shown that *Lactobacillus *strains play an effective role in protection of host against UTI (35). For instance, in 2001 it has been reported that *L. rhamnosus* GR1 has very high ability to bind with epithelial cells especially in vaginal tract and is resistant to spermicidal agents; therefore, the bacterium can prevent binding and growth of urinary and intestinal pathogens. In the present study, effect of three species of *L. casei* ATCC 32392, *L. rhamnosus* ATCC 7469 and *L. acidophilus* ATCC 4356 on growth level of urinary tract pathogens was investigated. Results showed that the size of inhibition zone related to *L. casei* ATCC 39392 was larger than other two species but the difference was not significant (*P*<0.05). These results were also confirmed by MIC test. Therefore, it can be concluded that *L. casei* ATCC 39392 is more effective compared to *L. rhamnosus* ATCC 7469 and *L. acidophilus* ATCC 4356. In most of the cases, there was not any considerable difference between susceptibility of *E. coli* isolates and the standard *E. coli* strain. According to other study there is a remarkable correlation between lactic acid production of *lactobacilli* under microaerophilic condition and their antibacterial effect which is probably due to effective leakage of hydrogen ion through cell membrane, and thus acidifying the cytoplasm. Accordingly, in this study, there was an indirect relationship between pH value of lactobacilli supernatants and their antibacterial effect that was confirmed by consideration of antibacterial effect of neutralized supernatants. In one study at 2000 it was found that lactic acid produced by *lactobacillus *strains can increase the susceptibility of Gram-negative bacteria to the antimicrobial agents (38). However, the results of our research showed no differences in the amount of MICs of AM, CP and cotrimoxazole between *E. coli* isolates before and after treating the bacteria with *lactobacilli* supernatants. Thus, it can be deduced that *lactobacilli* supernatants were not able to change the antibiotic resistance patterns of *E. coli* strains (38). 

Identification of the resistance mechanisms and their genetic origin can have a positive effect on production of new and/or more efficient antimicrobial agents. Antibiotic resistance in bacteria is generally due to existence of resistance genes on the plasmids. Plasmids are not essential for cell survival, but in special conditions like exposure to antimicrobial agents, can create resistance and thus guarantee the cell survival (33). The main types of responsible genes for resistance to beta-lactam antibiotics like OXA, SHV and TEM, are carried by plasmid. TEM1 is one of the most common beta-lactamases in *Enterobacteriaceae* family which is responsible for ampicillin resistance in 5% of isolated *E. coli* strains (39). Resistance to ampicillin in normal flora is mainly due to acquisition of TEM1 gene which exists on different plasmids (40). Studying the prevalence of antibiotic resistance genes among commensal *E. coli* strains is a useful criterion for discovering the existence of resistance genes in society (41). Since *E. coli* strain being isolated from urine in the present study, may have fecal origin, it was expected that resistance genes in isolated *E. coli* strains might be carried on plasmids inside the bacteria. Therefore, to investigate this issue, plasmid curing tests were carried out using ethidium bromide. As a result, plasmid deletion was detected in strain number 5. It should be noted that we observed some strains in replica plating tests that had lower growth than the maternal strains and thus produced smaller colonies on the medium. Plasmid patterns of these strains were also investigated and no differences were observed between plasmid patterns of the mentioned strains and the maternal strains. Therefore, it can be stated that abnormal colony size is due to a decrease in the number of plasmid copies not their complete deletion. Regarding plasmid patterns analyses, the removed gene is probably related to ampicillin, although further investigations are needed for definitive proof.

## Conclusion

Bacterial caused urinary tract infection (UTI) is one of the most frequent infections in human populations. Drug resistance often occurs in bacteria following inappropriate use of antibiotics. In this study, isolation of bacterial agents in UTI was performed on chrome agar medium. Among isolated bacteria, *E. coli*, *Klebsiellae*, *Enterobacter* and *Enterococcus* had the most frequency. This study, like other studies carried out on probiotics by different researchers, suggests that it is more beneficial that we use probiotics as partial replacement or adjunct to antibiotic therapy to help treating multi drug resistant UTIs.
